# Characteristics of medial-lateral postural control while exposed to the external perturbation in step initiation

**DOI:** 10.1038/s41598-019-53379-9

**Published:** 2019-11-14

**Authors:** Yun-Ju Lee, Jing Nong Liang, Bing Chen, Alexander S. Aruin

**Affiliations:** 1 Department of Industrial Engineering and Engineering Management, National Tsing Hua University, Hsinchu, Taiwan; 2 Department of Physical Therapy, University of Nevada Las Vegas, Las Vegas, NV USA; 3 Arms + Hands Abilitylab, Shirley Ryan Abilitylab, Chicago, IL USA; 4 Department of Physical Therapy, University of Illinois at Chicago, Chicago, IL USA

**Keywords:** Rehabilitation, Outcomes research

## Abstract

Controllability of posture in the medial-lateral direction is critical for balance maintenance, particularly in step initiation. The objective of the current study was to examine  the effects of external perturbation and landing orientation on medial-lateral control stability in step initiation. Eleven young healthy participants stood on the force platform and waited for the instruction of taking a step while experiencing a pendulum perturbation applied at the lateral side of the right shoulder. Eight experimental conditions were conducted by two levels of step side (right or left), two levels of perturbation (with or without), and two levels of landing orientation (forward or diagonal). The center of pressure (COP), pelvic movements, and muscle activities were recorded and analyzed as the onset of COP and pelvic movement, the COP displacement, and cocontraction and reciprocal muscle activation pattern. The temporal events of COP and pelvic movement were not significantly different in all experimental conditions. However, COP and pelvic movement were significantly later in the diagonal condition. Most of the segments showed reciprocal muscle activation patterns in relation to the perturbation released time. Subsequently, all segments showed cocontraction muscle activation patterns, which was significantly affected by step side, perturbation, and orientation. The results suggest that how the CNS initiated a step was identical with the COP then pelvic movement. The outcome highlights the importance of external perturbation and foot landing orientation effects on postural adjustments, which may provide a different approach to help step initiation.

## Introduction

Step initiation is used to assess mobility performance between healthy and patient populations. It has been observed that older adults or individuals with Parkinson’s disease fail to execute  a leg swing when initiating a step^1^. Prior to executing  a swing in step initiation, the body’s weight has to be shifted from even distribution of its weight to a single loading leg. This sequential pattern was inferred from measurements of the center of pressure (COP) displacements and is usually divided into three components: COP moves to the initial swing leg and backwards, COP then moves to the initial stance leg, and finally COP moves forward until toe-off of the swing leg^2,3^. Changes in the COP displacement indicate that step initiation is a predictable voluntary perturbation to postural stability. The central nervous system (CNS) employs feedforward^4–6^ and feedback mechanisms^7,8^ to maintain and restore balance. The forward/backward COP displacement is caused by reciprocal activation between ventral and dorsal muscles of the shank segment. It is an anticipatory postural adjustment (APA) handling the perturbation in the anterior-posterior direction during step initiation from COP shifting backward on the stance leg until heel off of the swing leg^3,9^. Meanwhile, hip abductors activation of the swing leg and reciprocal activations of the stance leg are considered as APA regulations for the perturbation in the medial-lateral (ML) direction^9^. Maintaining balance in the ML direction is even more challenging due to decreases in a base of support (BOS) from two legs to one stance leg. Furthermore, foot landing orientation affects BOS, changes the COP trajectory when stepping laterally^1,10^ and is also associated with slipping outcomes^11^. Pertinent to the current study, inappropriate COP displacement in the ML direction during step initiation has been observed in Parkinson’s disease^12^.

The lateral perturbation brought about by external impact causes side-specific postural control between the perturbed and contralateral side^13,14^. The correlated co-contraction and reciprocal activation of the trunk, thigh, and shank segments suggest that the CNS can modify postural muscle activation according to the perturbed and contralateral side of the body^13^, or a need to maintain body stability in the ML direction^14^. Corresponding postural control is also shown in the smaller COP displacements in the ML direction in response to the perturbation^14,15^. Together, step initiation or the lateral perturbations of the previous studies were all imposed as a single perturbation to balance maintenance. Effects of a single perturbation on postural adjustments have been well documented. Changes in muscle activities and COP are used to represent corresponding postural adjustments in response to the internal or external perturbations. When handling two perturbations simultaneously, instead of compromising the acceleration of arm flexion movement, the CNS recruits additional postural muscle activity to stabilize posture and reduce the COP displacement for the translational perturbation from the lower limb^16,17^. This indicates that strategies are adjusted based on the level of challenge to posture and balance, i.e. based on the context.

The external perturbation (pendulum impact) in the anterior-posterior direction during step initiation was shown to be a side-specific muscle coordination associated with both asymmetric perturbation and corresponding stance or swing leg side^18^. In spite of disturbed balance in the anterior-posterior direction, capacity of controlling ML postural stability has been associated with falls in older adults^19,20^. Step initiation^1,19^ or the lateral perturbation^13,14^ threatens postural stability in the ML direction and alters COP displacement. However, the external pulling system at the waist level could potentially improve step initiation in older adults^21^ and Parkinson’s patients^22^. This implies that the lateral perturbation might either assist or hinder movement depending on the impact of the swing or stance leg during step initiation. The objective of the current study was to investigate how medial-lateral perturbation (a pendulum would impact at the shoulder level on the right side of the body) affect postural muscles activity and COP in step initiation. We first hypothesized that muscle activity would be affected and modified accordingly, when taking a left or right step when a lateral perturbation applied at the right shoulder. Secondly, changes in foot landing orientation might affect the COP displacement and postural muscle activity of shank, thigh, and trunk segments.

## Methods

### Participants

Eleven young volunteers (5 males, 6 females, age = 28.09 ± 4.35 years, height = 1.67 ± 0.07 m, mass = 71.09 ± 18.75 kg) with the right dominant legs participated in the experiment. All participants were free from any musculoskeletal disorder and neurologic disease that could affect performing the experimental tasks. The project was approved by the Institutional Review Board at the University of Illinois at Chicago I All participants provided written informed consent before taking part in the experimental procedures. The experiment was performed in accordance with relevant guidelines and regulations.

### Procedure and instrumentation

**Figure 1:**
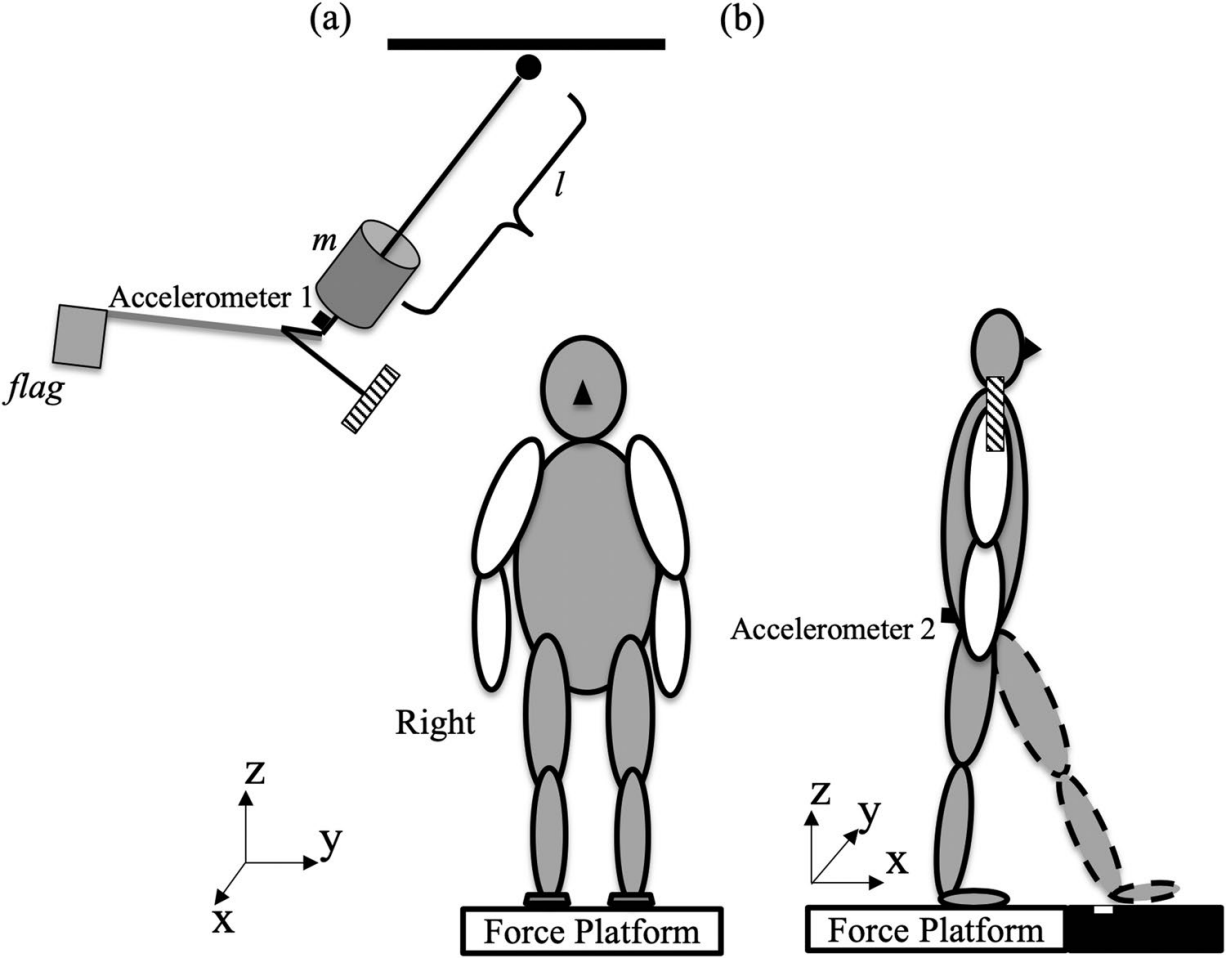
Experimental settings. Schematic representation of the experimental setup. Perturbations were induced by a pendulum impact applied to the right shoulder. A white straight-line mark was attached on a black platform, which was the same height with the force platform. *l* – is the adjusted height according to individual shoulder level. m – is the additional weight (5% BW) attached to the pendulum. 1- accelerometer attached to the pendulum, 2- accelerometer attached at the L5S1 level. (**a**) the original position from the front view(**b**) the end position in the left foot step condition from the lateral view.

The participants were instructed to stand still with their feet shoulder width apart and even body weight distribution between the left and right foot to the best of their effort on a force platform and waited for the experimenter’s instruction. Feet position was marked on the force platform to reproduce across the trials. A pendulum with a 30 cm long wooden stick and a flag was used to induce perturbations at the shoulder level on the right side of the body and allowed the participants to see the approaching pendulum via peripheral vision without a need for head rotation shown in Fig. 1^18^. The length of the central rod of the pendulum was adjusted to each individual’s shoulder height. A load (5% of the individual’s body mass) was attached to the pendulum next to its distal end. The pendulum was positioned at an initial angle of 30° to the vertical (0.8 m from the body) and was released by an experimenter 1 to 2 seconds after the start of each trial data collection. Perturbation consisted of unidirectional force applied by the pendulum to the lateral side of the right shoulder. The experimenter would then give the instruction to move the right or left foot forward or diagonal (45 degree outward) to step on the white straight-line mark. The distance between the original standing position to the white straight-line mark was adjusted to each individual’s step length. Two practice trials of initiating a step with the pendulum impact were performed prior to data collection. There were eight conditions (left or right foot, diagonal or forward step, and with or without perturbation). Three trials were collected in each condition. The order of the conditions was randomized across participants.

Two accelerometers were used in the experiment. The pendulum’s accelerometer (model 208CO3, PCB Piezotronics Inc, USA) was attached to the pendulum and its signal was used to determine the timing of the pendulum released and impact. The accelerometer worn by the participant (model 1356a16, PCB Piezotronics Inc, USA) was attached to the dorsal surface of the participant at the level of L5S1 to detect the temporal events of the pelvic movement. Ground reaction forces and moments of forces were recorded using the force platform (Model OR-5, AMTI, USA). EMG of muscles were recorded using disposable surface electrodes (Red Dot, 3 M, USA). After cleaning the skin with alcohol prep pads, the electrodes were attached to the bilateral muscle bellies of tibialis anterior (TA), medial gastrocnemius (MG), rectus femoris (RF), biceps femoris (BF), gluteus medius (GM), external oblique (EO), rectus abdominis (RA), and erector spinae (ES). The selected muscles have been used in previous studies of step initiation^23,24^ and anticipatory and compensatory postural adjustments involving externally induced body perturbations^15^. The placement of electrodes was based on recommendations reported in the literature^25^ and the interelectrode distance was 2 cm. EMG signals were band-pass filtered (10–500 Hz) and amplified (gain 2000) using the EMG system (Myopac, RUN Technologies, USA). The accelerometer signals, forces, moments of forces, and EMGs were synchronized and digitized with a 16-bit resolution at 1,000 Hz by means of an analog-to-digital converter and customized LabVIEW 8.6.1 software (National Instruments, USA).

### Data processing

All data were processed offline using MATLAB software (MathWorks, Natick, MA, USA). The signal from the pendulum’s accelerometers was used to determine the time of pendulum release (T_release_) following the verbal command and the time of impact (T_impact_). The signal from the accelerometer worn by the participant was used to detect the timing of the movement onset that the pelvic started to move in the up-down (Pelvicy_X-onset_), the left-right (Pelvic_Y-onset_), and the forward-backward (Pelvic_Z-onset_) directions. The onsets of the accelerometer signals were detected using the Teager-Kaiser onset time detection method^26^. The ground reaction forces and the moments of forces were filtered with a 20 Hz low-pass, 2^nd^ order, zero-lag Butterworth filter. Time-varying COP traces in the anterior-posterior and medial-lateral directions were calculated using the approximations described in the literature^27^. The onset of the COP moved away from the baseline was detected by the Teager-Kaiser method (COP_AP-onset_ and COP_ML-onset_). For magnitudes of the COP displacement, two posterolateral COP points were identified on the swing and stance legs, respectively. The first posterolateral COP on the swing leg was determined as COP_ML1_ in the ML direction and COP _AP1_ in the AP direction. The second posterolateral COP on the stance leg was determined as COP_ML2_ in the ML direction and COP _AP2_ in the AP direction. The first distance was compared to the baseline point (0, 0) and calculated as COP_ML1-distance_ (COP_ML1_ -0) and COP_AP1-distance_ (COP_AP1_ -0). The second distance between two posterolateral points was also calculated as COP_ML2-distance_ (COP_ML2_ -COP_ML1_) and COP_AP2-distance_ (COP_AP2_ -COP_AP1_). All variables were calculated for each trial and averaged over three trials.

All EMG data were high-pass filtered at 20 Hz, full-wave rectified, and low-pass filtered as linear envelope at 2 Hz (2^nd^ order Butterworth)^28^. Subsequently, the Teager-Kaiser method was used to detect the onset of muscle activity for individual muscle (EMG_onset_). The integrals of EMG activity of all studied muscles (∫EMGs) were calculated during the four phases: 1) 50 ~ 250 ms, 2) 250 ~ 450 ms, 3) 450 ~ 650 ms, and 4) 650 ~ 850 ms in relation to T_release_ (0 ms). These selected four phases were according to 50 ms of the postural response to the instruction cue^29^, a 200 ms window of postural adjustment evaluations^4,30^ and COP shifting between the stance and swing legs during step initiation^3^. Moreover, ∫EMGs of the baseline activity during the 200 ms time window (0–200 ms) were obtained at the beginning of the trial.

Two approaches were used to evaluate changes in muscle activity during each phase. One approach was to eliminate effects of each muscle baseline activity by reduction of ${\int }^{}baseline$; as a result, activation of muscles was described by values larger than zero (∫EMG > 0) and inhibition by values smaller than zero (∫EMG < 0). Thus, the ∫EMG were normalized by ∫EMGmax, which was the maximum value throughout all experimental trials for each muscle for each phase, separately as:

$${\int }^{}baseline={\int }_{0}^{200}EMG$$

$${\int }^{}EM{G}_{50 \sim 250}=\frac{{\int }_{{T}_{release}+50}^{{T}_{release}+250}EMG-{\int }^{}baseline}{{\int }^{}EMGmax},$$

$${\int }^{}EM{G}_{250 \sim 450}=\frac{{\int }_{{T}_{release}+250}^{{T}_{release}+450}EMG-{\int }^{}baseline}{{\int }^{}EMGmax},\,and\,etc.$$

Subsequently, the second approach was based on calculations of the sums and differences between normalized ∫EMG values of ventral and dorsal muscles for the shank, thigh and trunk in each phase, separately.

$$\,{\rm{C}}={\int }^{}EMGventral+{\int }^{}EMGdorsal,\,{{\rm{R}}=\int }^{}EMGventral-{\int }^{}EMGdorsal$$

C indexes were calculated as the sum of the integrals of activity of the antagonist-agonist muscle pairs to represent co-activation and R indexes as the difference between the integrals of muscle activity in the muscle pairs to represent reciprocal activation^31^.

Thus, the C and R values for the shank segment in the 50~250 phase were calculated as:

$$\begin{array}{rcl}{C}_{shank50 \sim 250} & = & {\int }^{}T{A}_{50 \sim 250}+{\int }^{}M{G}_{50 \sim 250}\\ {R}_{shank50 \sim 250} & = & {\int }^{}T{A}_{50 \sim 250}-{\int }^{}M{G}_{50 \sim 250}\end{array},$$

All variables were calculated for each trial and averaged over three trials.

### Statistics

A three-way repeated-measures ANOVA were performed with three factors: step (2 levels: right step and left step), perturbation (2 levels: with and without perturbation) and orientation (2 levels: forward and diagonal) on the onset and EMG integrals of four phases for individual muscles.

A two-way repeated measures ANOVA was performed with two factors: step (2 levels: right step and left step) and orientation (2 levels: forward and diagonal) on a duration between T_release_ and T_impact_. Three-way repeated measures ANOVA was performed with three factors: step (2 levels: right step and left step), orientation (2 levels: forward and diagonal), and perturbation (2 levels: with and without perturbations) on temporal variables (Pelvic_X-onset_, Pelvic_Y-onset_, Pelvic_Z-onset_, COP_AP-onset_, and COP_ML-onset_), and on the COP distances (COP_ML1-distance_, COP_AP1-distance_, COP_ML2-distance_, and COP_AP1-distance_).

In addition, identifications of either cocontraction (C) or reciprocal (R) activation pattern were employed in each phase for each segment. The EMG integrals of muscle coupling for the shank, thigh, and trunk segments were compared using C and R values. Paired sample t tests were used to determine that the significantly larger C values compared to R values indicated the presence of co-contraction in a segment. When ventral and dorsal muscles were activated (larger than the baseline), calculations from both positive values of ventral and dorsal muscles revealed higher C value than R value and vice versa. If R values were significantly larger as compared to C values, this would indicate a prevalence of reciprocal activation^13^. Subsequently, if a muscle coupling of the shank, thigh and trunk segments had C value larger than R value, three-way repeated measures ANOVAs were performed with three factors: step (2 levels: right step and left step), orientation (2 levels: forward and diagonal), and perturbation (2 levels: with and without perturbations) to evaluate the C rather than R, and vice versa.

Post hoc comparisons were performed using Tukey’s Honest Significant Difference test for significant interactions. Statistical difference was set at *p* < 0.05. Means and standard errors are presented in the results and figures.

## Results

**Figure 2:**
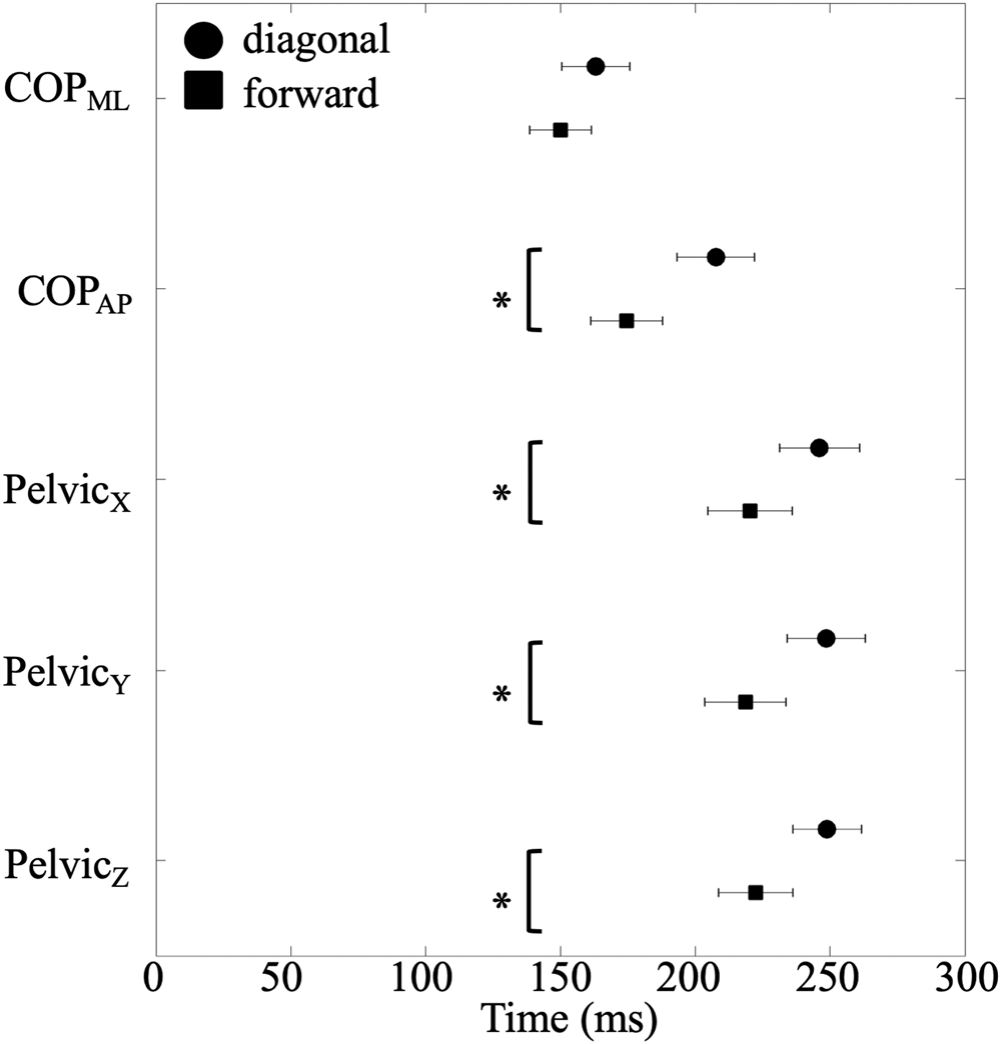
Sequential events of COP and pelvic movement. Onset times of center of pressure (COP) in the medial-lateral (ML) and anterior-posterior (AP) directions, three-dimensional accelerations in the up-down (Pelvic_X-onset_), the left-right (Pelvic_Y-onset_), and the forward-backward (Pelvic_Z-onset_) directions. Circles represent the diagonal conditions and squares represent the forward conditions. The time 0 indicates the moment of the pendulum released. Asterisks indicate significant differences between the diagonal and forward conditions (p < 0.05).

The duration between T_release_ and T_impact_ was not significantly affected by the factors of step and orientation. The duration was not different across four conditions (2 level steps and 2 level orientation) and the mean duration was 552.11 ± 11.01 ms. Furthermore, the data averaged across eight conditions (2 level steps, 2 level orientation and 2 level perturbations) and presented in the temporal sequence was that COP_ML-onset_ at 156.60 ± 11.57 ms and followed by COP_AP-onset_ at 191.01 ± 13.52 ms, Pelvic_X-onset_ at 233.17 ± 14.87 ms, Pelvic_Y-onset_ at 233.57 ± 13.29 ms, and Pelvic_Z-onset_ at 235.61 ± 13.08 ms in relation to T_release_. Repeated measures ANOVA revealed that the factor of orientation significantly affected the onset time of both COP and body movement (Table 1). Figure 2 illustrates that COP and pelvic movement initiated slower in the diagonal conditions than the forward conditions, except COP_ML-onset_.

**Table 1:** The results of two-way repeated measure ANOVAs for COP_AP-onset_, COP_ML-onset_, Pelvic_X-onset_, Pelvic_Y-onset_, and Pelvic_Z-onset_.

	Step (S)	Orientation (O)	Perturbation (P)	S × O	S × P	P × O	S × O × P
	F(1,10) (*p*)	F(1,10) (*p*)	F(1,10) (*p*)	F(1,10) (*p*)	F(1,10) (*p*)	F(1,10) (*p*)	F(1,10) (*p*)
COP_AP-onset_	4.188 (0.068)	32.541 (**<0.001**)	1.600 (0.235)	0.257 (0.623)	0.012 (0.914)	0.283 (0.606)	2.634 (0.136)
COP_ML-onset_	0.143 (0.714)	3.607 (0.087)	0.587 (0.461)	1.968 (0.191)	0.587 (0.461)	0.702 (0.422)	0.807 (0.390)
Pelvic_X-onset_	0.054 (0.821)	14.296 (**0.004**)	2.598 (0.138)	4.305 (0.065)	0.810 (0.389)	0.098 (0.760)	2.394 (0.153)
Pelvic_Y-onset_	0.439 (0.523)	5.249 (**0.045**)	0.113 (0.744)	0.108 (0.749)	0.168 (0.690)	1.206 (0.298)	0.269 (0.615)
Pelvic_Z-onset_	1.384 (0.267)	33.139 (**<0.001**)	0.372 (0.555)	6.212 (**0.032**)	0.012 (0.916)	0.676 (0.430)	0.423 (0.530)

Significant p-values are shown in bold.

**Figure 3:**
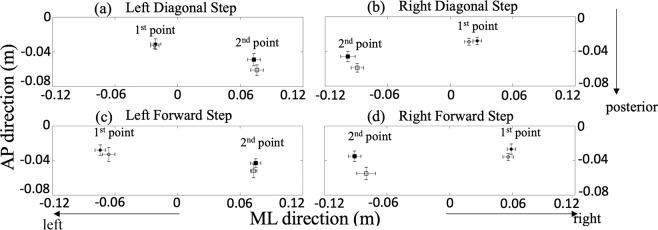
COP positions of the first and second posterolateral points. Circles represent the positions of COP_ML1_ with COP_AP1_ and squares represent the positions of COP_ML2_ with COP_AP2_ in the with (white) and without (black) perturbations in the left diagonal step condition (**a**), the right diagonal step condition (**b**), the left forward step condition (**c**), and the right forward step condition (**d**).

The two representative points of COP, the first posterolateral COP on the swing leg (COP_ML1_ and COP_AP1_) and the second posterolateral COP on the stance leg (COP_ML2_ and COP_AP2_), are illustrated in Fig. 3 for all experimental conditions. In the left step conditions, COP moved towards to the left side (1^st^ point in Fig. 3A,C) then shifted towards to the right side (2^nd^ point in Fig. 3A,C). In the right step conditions, COP moved towards to the right side (1^st^ point in Fig. 3B,D) then shifted towards to the left side (2^nd^ point in Fig. 3B,D). Overall averaged cross the forward, diagonal, with, and without perturbation conditions, COP_ML1_ was 0.045 ± 0.009 m, COP_AP1_ was −0.031 ± 0.006 m, COP_ML2_ was −0.074 ± 0.005 m, and COP_AP2_ was −0.051 ± 0.006 m. COP showed the opposite direction in the right step conditions, COP_ML1_ was −0.040 ± 0.007 m, COP_AP1_ was −0.030 ± 0.004 m, COP_ML2_ was 0.089 ± 0.007 m, and COP_AP2_ was −0.049 ± 0.007 m.

**Figure 4:**
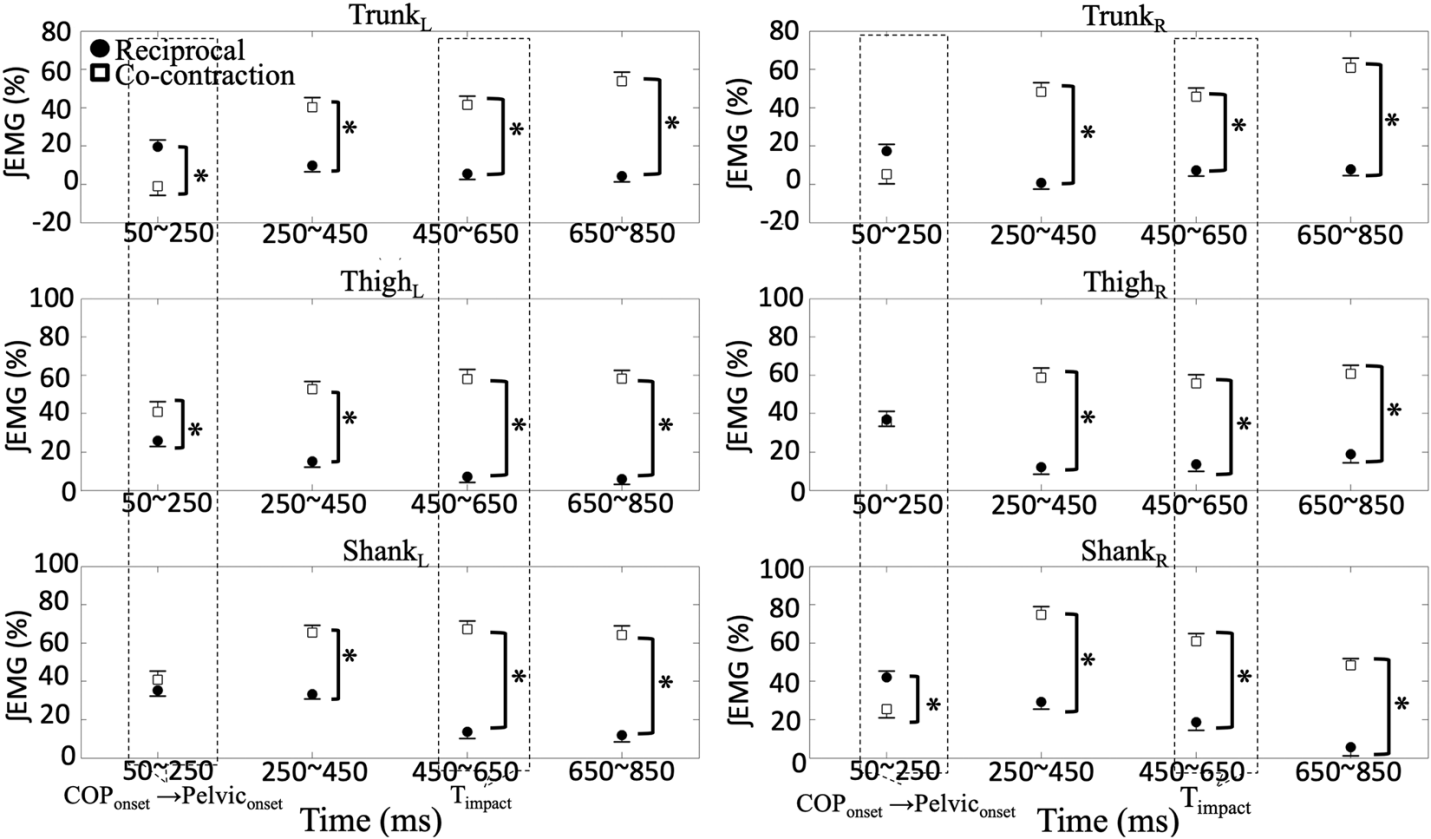
C and R values of three segments. Averaged C and R values through all experimental conditions for the trunk, thigh, and shank segments on both, left (L) and right (R) sides calculated during the 50~250, 250~450, 450~650, and 650~850 phases. Circles represent R values and squares represent C values. Asterisks indicate significant differences between C and R values for each segment revealed by paired sample t tests (p < 0.05).

In addition, Fig. 4 shows the C and R values in all the experimental conditions of each segment for each phase. First, the patterns of muscle co-contraction (C) and reciprocal (R) activation were identified for each segment. In the 50~250 ms phase, similar R and C values for the Trunk_R_ and Thigh_R_ segments and the Shank_L_ segment were observed. While, significant larger R values in the Shank_R_ and Trunk_L_ segments with a significant larger C value in the Thigh_L_ segment were observed. In the subsequent phases, significant larger C values were observed for all segments compared to R values. The large C values indicate co-contraction of muscle in all segments after 250 ms in relation to T_release_.

**Figure 5:**
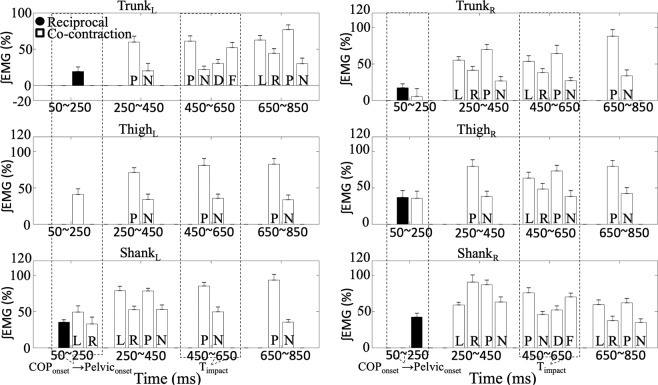
Effects of three factors on C and R values. Comparisons of step (L: left v.s. R: right), orientation (D: diagonal v.s. F: forward), and perturbation (P: with v.s. N: without) were illustrated. Black bars represent R values and white bars represent C values. The dash boxes indicate the occurrences of overall COP, Body movement, and the timing of external perturbation impacted (T_impact_).

Further examinations of co-contraction and reciprocal activation of muscles were conducted by three-way repeated measures ANOVA and revealed significant main effects on all segments in the four phases (Table 2). The factor of step significantly affected C values on the Shank_L_ segment (50~250 ms phase and 250~450 ms phase), Shank_R_ segment (250~450 ms phase and 650~850 ms phase), Thigh_R_ segment (450~650 ms phases), Trunk_L_ segment (650~850 ms phase), and Trunk_R_ segments (250~450 ms phase and 450~650 ms phases). Figure 5 illustrates the effects of three factors and letters within the bars represent the corresponding experimental conditions for the significant main effect of the factor. The larger C values were observed in the left step conditions than the right step conditions, except the Shank_R_ segment in the 250~450 ms phase. Additionally, the factor of perturbation significantly affected C values of bilateral segments in the 250~450 ms, 450~650 ms, and 650~850 ms phases. All C values were significantly larger in the with perturbation conditions than the without perturbation conditions. The factor of orientation significantly affected C values on the Trunk_L_ and Shank_R_ segment in the 450~650 ms phase and showed the larger C values in the forward condition than the diagonal conditions.

**Table 2:** The results of three-way repeated measure ANOVAs for the C and R values of left and right trunk, thigh, and shank segments in the 50~250, 250~450, 450~650, and 650~850 phases.

		Step (S)	Perturbation (P)	Orientation (O)	S × P	S × O	P × O	S × P × O
		F(1,10)	F(1,10)	F(1,10)	F(1,10)	F(1,10)	F(1,10)	F(1,10)
Left								
Trunk	R_50~250_	2.225	0.319	0.153	2.368	1.406	0.573	0.033
	C_250~450_	7.63	34.680*	1.115	0.146	1.491	2.951	0.471
	C_450~650_	4.081	20.128**	5.605*	2.192	0.288	3.240	0.088
	C_650~850_	6.346*	22.511**	3.756	2.105	7.306*	2.911	3.350
Thigh	C_50~250_	0.863	1.818	0.025	1.107	0.067	2.123	1.964
	C_250~450_	0.614	42.642**	0.806	0.012	2.201	0.041	0.337
	C_450~650_	3.590	28.564**	0.107	3.248	0.750	0.077	2.787
	C_650~850_	4.054	42.138**	3.733	1.700	0.962	0.464	1.478
Shank	C_50~250_	5.146*	2.672	0.034	1.748	0.076	0.918	0.969
	R_50~250_	1.497	1.784	1.237	0.076	0.044	5.447*	2.674
	C_250~450_	13.898**	24.468**	0.014	0.000	0.680	0.002	0.967
	C_450~650_	0.290	28.954**	3.609	8.117*	5.151*	0.163	0.001
	C_650~850_	0.961	34.252**	0.652	0.602	0.004	0.106	4.706
Right								
Trunk	C_50~250_	0.882	0.414	0.485	0.835	8.025*	0.087	8.380*
	R_50~250_	0.079	0.003	3.145	0.402	0.285	0.073	0.048
	C_250~450_	12.230**	22.271**	0.009	0.028	0.020	1.363	1.175
	C_450~650_	9.252*	11.571**	0.882	0.009	3.098	0.883	1.129
	C_650~850_	1.856	60.296**	0.316	7.292*	0.684	0.005	0.096
Thigh	C_50~250_	0.891	2.824	2.469	0.209	0.014	0.002	2.094
	R_50~250_	0.040	2.665	9.316*	0.174	0.217	0.081	0.000
	C_250~450_	4.567	40.369**	2.500	1.018	8.852*	0.384	7.926*
	C_450~650_	6.420*	41.473**	3.352	4.068	0.025	1.693	0.056
	C_650~850_	1.577	52.387**	1.059	7.428*	0.062	0.001	0.505
Shank	R_50~250_	0028	2.533	2.003	0.086	0.158	0.152	0.041
	C_250~450_	11.674**	8.107*	0.004	0.677	0.1450	0.008	0.376
	C_450~650_	0.074	18.372**	8.029*	0.581	6.618*	5.246*	0.506
	C_650~850_	6.888*	24.978**	0.158	0.386	1.261	0.957	1.237

*p < 0.01; **p < 0.001.

## Discussion

The current study is the first to investigate how external-lateral perturbations and landing orientation affect the COP displacement in left or right step initiation. The sequence of events observed were: COP_ML-onset_, then COP_AP-onset_, followed by Pelvic_X-onset_, Pelvic_Y-onset_, and Pelvic_Z-onset_ with respect to T_release_. The factor of step affected the directional deviation of COP in the ML direction, not the AP direction. While the factor of orientation affected the onset time of COP in the AP direction and pelvic movement. The COP distances from the swing leg to the stance leg were affected by the factor of perturbation and orientation. The shorter COP distance was observed in the with perturbation condition and in the diagonal condition. Reciprocal activation patterns were observed on the left trunk and right shank segment in the 50~250 phase. After 250 ms in relation to T_release_, co-contraction patterns were observed and significantly affected by the factor of perturbation on bilateral shank, thigh, and trunk segments.

### Temporal events in step initiation

The synchronization timing was set at the time of the pendulum release (T_release_) and the given instruction. After T_release_, the initiations of COP then pelvic movement were observed in all experimental conditions. The little variability in terms of duration indicated that the participants were aware of and impacted by the pendulum almost around the same time in the perturbation conditions. The sequence of temporal events was the same as in previous studies^9,10^ and indicates that participants did not assume to make a left or right step before the instruction. In addition, the onset of COP in the ML then AP direction suggested the right or left step would be decided first and followed with body weight shifting on the swing leg in step initiation. Subsequently, the timing of the first and second posterolateral points were observed at 402.46 ± 28.40 ms and 686.86 ± 72.27 ms in relation to T_release_ averaged over all conditions, respectively. It was confirmed that the external-lateral perturbation was imposed (at 552.11 ± 11.01 ms) in the period of body weight shifting between the swing and stance leg during step initiation^2,3^.

### Effects of step

The non-significant different onset of COP and pelvic movement between the right and left step conditions indicated that participants expected instructions to move to the right or left step first equally. The first and second posterolateral points were observed on the left and right sides in the left step condition, respectively and vice versa in the right step condition (Fig. 3). It confirmed that COP first shifted on the swing leg then toward the stance leg^2,3^. In addition, the first and second COP distances in both directions were not different between the left and right step conditions. These similar points and distances might support the idea that participants indeed stood with even body weight distribution between the left and right foot at the beginning of the trials.

Side-specific differences in muscle activation patterns between left and right segments was not observed in all experimental conditions. However, the C values were shown to be different for bilateral segments between the right and left step conditions (Fig. 5 & Table 2). The framework of C- and R-commands controlling a segment to effectively change muscle co-contraction if moving the endpoint of a segment while R-command will be more pronounced with no or small effects on the endpoint in response to the perturbations^32,33^. When the significant larger C value was observed, it indicated that coupling muscles were both activated and participants utilize co-contraction strategy to increase stability for the perturbations^13,34^. Taking the right step condition as an example, the right shank segment was the swing leg and increased TA muscle activity for the first posterolateral COP distance^35,36^. This increased TA muscle activity was also used to prepare the forthcoming external lateral perturbation, which was anticipatory postural adjustment in relation to the perturbation^14^. Subsequently, in the perturbation impact phase, the larger C values were observed on the right thigh and shank segments in the left step condition. In the left step condition, right thigh and trunk segments were the stance side and increased muscle activities for loading weight^36^ as well as for the perturbation^14^. On the contrary in the right step condition, right segments were the swing side and activated fewer muscle activities compared to acting as the stance leg in the left step condition.

### Effects of the external-lateral perturbation

The external-lateral perturbation was imposed at the right shoulder level during the phase of weight shifting from the swing leg to the stance leg. The orientation of the COP trajectory and the perturbation were in the same direction in the right step conditions and showed the opposite direction in the left step conditions. When the left leg was the stance side (the right step condition), COP_ML2-distance_ was smaller in the with perturbation condition (0.121 m) compared to the without perturbation condition (0.136 m). It might be comparable with the waist-pulling cable system for step initiation and showed improvements of decreasing weight transfer time in older adults^21^ and Parkinson’s patients^22^ along with the pulling direction. Decreases in the horizontal distance has been observed in older adults with Tai Chi exercise and showed improvements of effective postural stability^2^. Similarly, the perturbation in the current study was like a push to help to shorten the ML COP distance in the right step condition. In the left step condition, COP_ML2-distance_ was around 0.119~0.122 m regardless of the occurrence of the perturbations and smaller than the right step condition. When the right leg was the stance leg, the time of COP moved to the stance leg was close to the time of perturbation impact. Hence, participants attentively pulled themselves away from the side of the potential forthcoming perturbation and resulted in the same effects of the perturbation directly impacted the body.

In the current study, participants might expect the forthcoming pendulum due to it was released while the instruction but not actually impacted at the shoulder level in the without perturbation condition. Bilateral segments showed the same muscle activation patterns in the without and with perturbation conditions and acted as the early postural adjustment to the external-lateral perturbation^32,37^. Once participants were aware that the trial was without the perturbation, the C values of all segments were significantly smaller compared to the with perturbation condition. The muscle activities in the without perturbation condition represented their performance for step initiation and differences between two conditions indicated additional muscle activities for handling the external-lateral perturbation. The current outcomes supported the first hypothesis that the CNS increased corresponding muscle activities with cocontraction in the APA and CPA phase in relation to the predictable and asymmetrical perturbation^13,14^.

### Effects of landing orientation

There was a significant difference in onset of COP and pelvic movement between the forward and diagonal conditions, except COP in the ML direction. After initiating the COP displacement on the swing leg, COP shifted further lateral on the swing leg when intending to land the step diagonally. It was not only reflected by the position of COP_ML1_ (Fig. 3) but also the magnitude of COP_ML1-distance_. It was comparable with the ML displacement of the S1 period^1^ with a shorter distance while COP reached to the first posterolateral point faster in the diagonal condition. Meanwhile, the shank and trunk segments showed less co-contraction in the diagonal condition. Together this may indicate that the CNS require more time to prepare COP movement and reduce muscle contractions for step landing diagonally. It was supported by the fact that young adults showed further ML COP displacement on the swing leg and finally making a quick step^38^ as well as the larger step widths observed when balance is challenged by external perturbations^39^. Foot placement controllability in pathological gait^40^ might also explain that difficulties of diagonal step initiation during the postural phase in Parkinson’s disease^41,42^.

## Conclusions

In step initiation, the sequence of temporal events is from standing, COP shifts in ML direction to determine the swing and stance legs, and moves backward then following with pelvic movement. This sequence is consistent with the step side as well as landing orientation and not compromised by external perturbations. Instead, the COP distance and muscle co-contraction levels of segments were adjusted according to the appearance of perturbations and landing orientation to enhance postural stability. Control of step initiation through muscle coordination and foot placement might be benefits to individuals with gait stability difficulties and provide different approaches for developing rehabilitation programs.
